# Potential of Lyophilized Platelet Concentrates for Craniofacial Tissue Regenerative Therapies

**DOI:** 10.3390/molecules26030517

**Published:** 2021-01-20

**Authors:** Nurul Aida Ngah, Jithendra Ratnayake, Paul R. Cooper, George J. Dias, Darryl C. Tong, Siti Noor Fazliah Mohd Noor, Haizal Mohd Hussaini

**Affiliations:** 1 Faculty of Dentistry, Sir John Walsh Research Institute, University of Otago, P.O. Box 56, Dunedin 9054, New Zealand; ngaai159@student.otago.ac.nz (N.A.N.); p.cooper@otago.ac.nz (P.R.C.); darryl.tong@otago.ac.nz (D.C.T.); haizal.mh@otago.ac.nz (H.M.H.); 2 Department of Oral Sciences, Faculty of Dentistry, University of Otago, P.O. Box 56, Dunedin 9054, New Zealand; 3 Department of Anatomy, School of Biomedical Sciences, University of Otago, P.O. Box 56, Dunedin 9054, New Zealand; george.dias@otago.ac.nz; 4 Craniofacial and Biomaterial Sciences, Advanced Medical and Dental Institute, Universiti Sains Malaysia, Bertam, Kepala Batas 13200, Malaysia; fazliah@usm.my

**Keywords:** lyophilization, platelet concentrate, platelet-rich fibrin, craniofacial regeneration, tissue engineering

## Abstract

Objective: The use of platelet concentrates (PCs) in oral and maxillofacial surgery, periodontology, and craniofacial surgery has been reported. While PCs provide a rich reservoir of autologous bioactive growth factors for tissue regeneration, their drawbacks include lack of utility for long-term application, low elastic modulus and strength, and limited storage capability. These issues restrict their broader application. This review focuses on the lyophilization of PCs (LPCs) and how this processing approach affects their biological and mechanical properties for application as a bioactive scaffold for craniofacial tissue regeneration. Materials and Methods: A comprehensive search of five electronic databases, including Medline, PubMed, EMBASE, Web of Science, and Scopus, was conducted from 1946 until 2019 using a combination of search terms relating to this topic. Results: Ten manuscripts were identified as being relevant. The use of LPCs was mostly studied in in vitro and in vivo craniofacial bone regeneration models. Notably, one clinical study reported the utility of LPCs for guided bone regeneration prior to dental implant placement. Conclusions: Lyophilization can enhance the inherent characteristics of PCs and extends shelf-life, enable their use in emergency surgery, and improve storage and transportation capabilities. In light of this, further preclinical studies and clinical trials are required, as LPCs offer a potential approach for clinical application in craniofacial tissue regeneration.

## 1. Introduction

Platelet concentrates (PCs) typically refer to a group of materials produced from autologous blood designed to improve tissue regeneration [1]. PCs were initially created as fresh plasma preparations for autologous application as a regenerative scaffold to enable optimal growth factor release [2]. Several terms have been used for PCs according to their final form used, including plasma-enriched platelets (PEPs), platelet-rich plasma (PRP), platelet releasate (PR), preparation rich in growth factor (PRGF) [1], platelet-rich fibrin (PRF) [2], and concentrated growth factor (CGF) [3]. They provide the potential for simultaneous delivery of an abundance of bioactive molecules, including transforming growth factor beta-1 (TGF-β1), platelet-derived growth factor (PDGF), vascular endothelial growth factor (VEGF), epidermal growth factor (EGF), and insulin-like growth factor (IGF) (Kobayashi et al., 2017). These PC preparations have been established as an affordable, safer and more potent way to release high concentrations of cocktails of growth factors locally for enhanced tissue regeneration [4].

Marx [5] published the initial report on the first-generation of PCs as PRPs. These PCs were used as bone substitutes to treat osseous defects in a variety of areas of dentistry, including oral and maxillofacial surgery, periodontology, and plastic surgery [5,6]. Despite their early promise, several drawbacks of PCs were identified mainly as a result of the inclusion of anticoagulants and bovine thrombin [7].

PRF is a second-generation PC, which offers the benefit of being synthesized without the need for anticoagulants or other artificial by-products that inhibit the coagulation cascade. PRF is autologous and naturally derived and meets all three essential tissue regeneration requirements including:

(a) Provision of a three-dimensional fibrin scaffold;
(b) Containing autologous cells;
(c) Functioning as a store of endogenous growth factors for release for up to 14 days [8].

Notably, this PRF has been found to improve the deposition and regeneration of bone [9]. In this review, the term PCs will be used for all types of platelet concentrates, such as PRP, PRF, PRGF, CGF, etc.

PCs have gained popularity in dentistry and craniofacial surgery as a therapeutic alternative for tissue regeneration. Jeon [10] performed a bone regeneration study using PCs in twenty-four New Zealand White rabbit cranial defect models. The research showed that bone regeneration is higher in PCs classes (collagen sponges without PCs). They observed that the PC was efficacious in bone regeneration and could be used as an adjunct therapy for bone regeneration. Moreover, Anitua [1] presented the biocompatibility of PC preparation, as this method has encouraged clinical use in regenerative therapies, particularly in oral and maxillofacial surgery. They emphasized that the emergence and progress of novel biomaterials can revolutionize medicine. The combination of platelet-rich preparations and biomaterials has increased the therapeutic value of this biomaterial. PC extract can bridge the gap between scaffolds and cell biology, adding the biologic stimulus required for functional tissue regeneration. Furthermore, PCs also can be used in the treatment of recurrent ulcers, as the cavity can be filled with activated PCs. permitting the fibrin matrix to form inside the ulcer bed.

However, PCs were designed for immediate and short-term use (for up to 14-days); thus, this approach somewhat limits their therapeutic value [11,12]. As longer periods are reportedly required for the enhancement of bone regeneration, more effective methods for the delivery of growth factors over longer time-periods have been sought [13]. Furthermore, PCs are processed from autologous blood prior to clinical use, and, therefore, their bioactivity can vary between individuals. Their low viscosity, elastic modulus and strength are also issues, which mean PCs are technically challenging to handle in the clinical setting. Finally, due to the requirement for the need for their use within a short time-frame, as they cannot be easily stored or transported, their application is not well suited for emergency care medicine [14]. These issues restrict the use of PCs for broader therapeutic applications [15].

Notably, it has recently been reported that in addition to improved durability, lyophilized PC (LPC) formulations provide better handling, storage, and transport capabilities. They are, therefore, now being preferred for large-scale production [16]. Several researchers have specifically reported protocols to enable the generation of LPCs which exhibit longer-term survival and preservation characteristics [17,18,19,20]. This review reports on how lyophilization approaches affect the handling and biological properties of PCs and identify their potential for application in craniofacial tissue regenerative medicine.

## 2. Results and Discussion

### 2.1. Overview of Lyophilization for Platelet Concentrates

Lyophilization, or freeze-drying, has been developed as a method for transforming solutions containing labile substances into more stable solids to enable their distribution as well as preserving bioactivity for different applications. This process is widely used to enhance the stability and long-term storage of proteins in the pharmaceutical, biotechnology and food industries [16]. Indeed, freeze-drying offers storage and processing benefits over conventional methods, including (1) longer storage times at room temperature; (2) rapid transformation by rehydration, which enables practical application in emergency medicine; and (3) increased stability to enable transport and application at distant sites [21,22].

**Figure 1:**
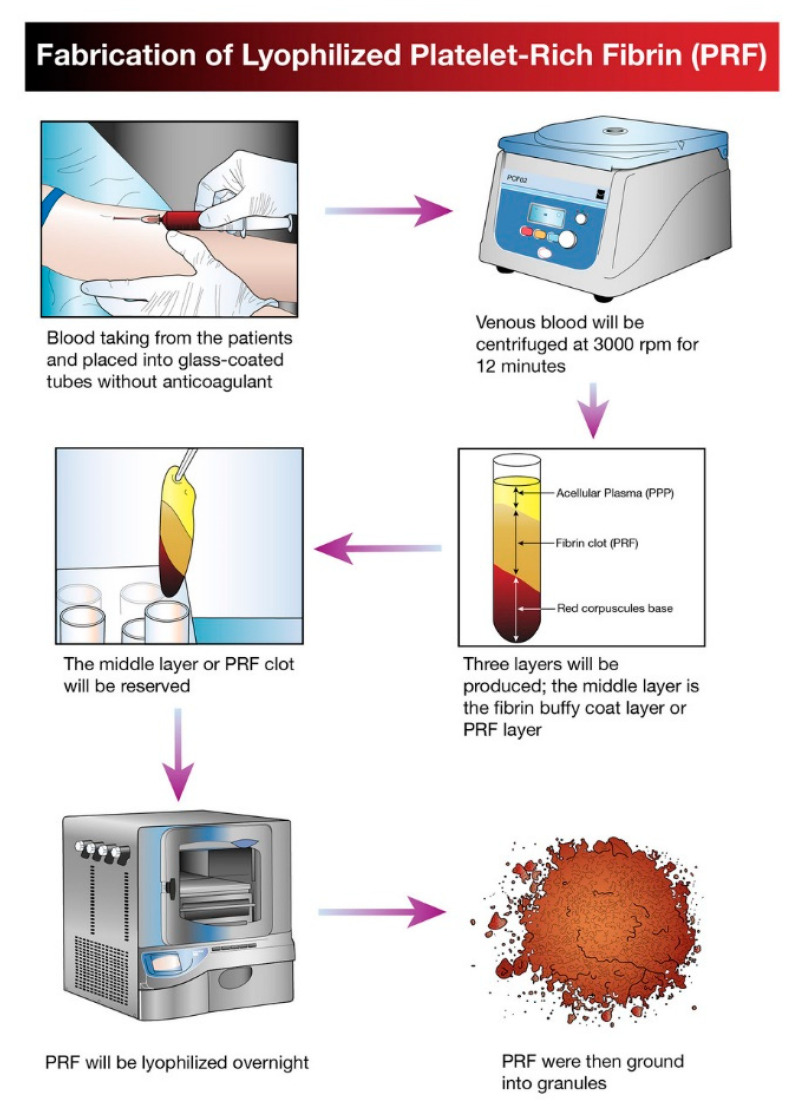
Schematic diagram illustrating the processing and development of lyophilized platelet concentrate (LPC) in the form of platelet-rich fibrin (PRF).

**Figure 2:**
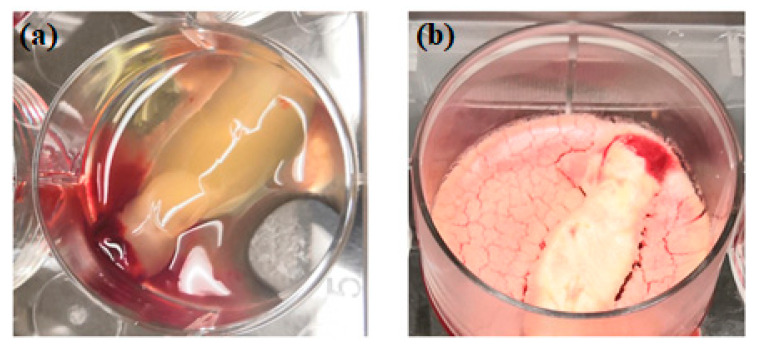
Physical comparison of traditionally prepared platelet concentrates (**a**) versus lyophilized platelet concentrates (**b**).

The idea of platelet lyophilization was originally proposed by Wolkers [23], who initially indicated that freeze-dried platelets functioned biologically similarly to endogenous platelets. Indeed, these lyophilized platelets not only exhibit increased storage stability but are also able to rapidly release bioactive growth factors, such as platelet-derived growth factor-BB (PDGF-BB), TGF-β1, and VEGF at the surgical site [24,25,26]. It does not have a detrimental effect on PCs’ ability to facilitate tissue regeneration, suggesting that a range of cytokines and fibrin networks in the PCs are preserved and have the capacity to promote chemotaxis and cell proliferation [27]. LPCs express their growth factors more slowly than PCs. This was discovered in a study by Zheng [13] when they attempted to combine nano-hydroxyapatite (nHA), L-lactic acid-co-glycolic acid (PLGA), and the hydrogel (nHA/PLGA/Gel) scaffold with LPCs. The in vitro release experiments showed that the composite scaffold allowed for gradual and continuous release of PRF-derived growth factors. They examine the pattern of growth factors released from the composite scaffolds over 12 weeks and illustrated that the concentration of IGF-I, TGF-β1, and PDGF-AB in the four weeks reached 66%, 67%, and 65%, respectively. It was demonstrated that there was a higher release rate in the first 4 weeks followed by a relatively steady rate over the following weeks. Figure 1 provides a schematic diagram of the method used for producing lyophilized PCs, and Figure 2 provides a physical comparison of PCs and lyophilized PCs.

The advantages of lyophilized platelet concentrates are further highlighted in a study that recently documented lyophilized PCs exhibiting enhanced osteogenic capability and improved tissue compatibility at the injury site, compared with freshly isolated PCs [17].

### 2.2. Lyophilized Platelet Concentrates in Craniofacial Tissue Regeneration

The use of PCs to deliver growth factors to defective areas to stimulate tissue regeneration in both the medical and dental fields has gained considerable interest over recent years. However, despite the considerable advantages offered by LPCs, only a limited number of studies, summarized below, have been reported. This review reports on the current application and potential of LPCs in craniofacial tissue regeneration. Table 1 provides details of studies reporting the different preparation methods of LPCs for potential use as craniofacial scaffolds for tissue regeneration.

Despite its excellent safety and efficacy, PCs suffer from several significant disadvantages that limit their broader clinical applications. Current drawbacks include the processing requiring open membrane handling and a reduced elastic modulus that does not support suturing. Recent research has attempted to address these issues. One recent analysis compared the biological and mechanical properties of fresh, frozen, and lyophilized PC membranes produced by the traditional bottle-tube approach and those produced in a singular-syringe closed system. The data demonstrated that a relatively small number of mesenchymal stem cells could attach to fresh PC membranes compared with those that were frozen or lyophilized [32]. Furthermore, frozen and lyophilized PCs exhibited a more compact structure with an uneven texture compared with fresh PCs, and this may explain the differences observed in the properties of the tensile strength reported [32]. In 2017, Nakatani [24] performed a bone engineering study on mice calvarias using an LPC product. The researchers showed preservation of biological properties of LPCs even after exposure to lyophilization, as they observed that the sum of PDGF-BB and TGF-B1 was maintained by lyophilization. They concluded that the LPC can increase cranial bone regeneration similar to the process observed in traditionally prepared PCs. They postulated that the biological function of growth factor during the bone healing period was preserved.

Periodontitis is an important disease in dentistry that can result in both hard and soft tissue loss. Periodontal regeneration and healing can be enhanced by utilizing growth factors that modulate cellular responses, and, therefore, LPCs have been incorporated into chitosan hydrogel scaffolds containing encapsulated periodontal ligament stem cells (PDLSCs) [28]. PDLSC proliferation and migration rate increased dramatically with LPC inclusion as compared with fresh PC-containing hydrogel controls. This outcome was reportedly potentially due to both the increased pore diameter in the LPC containing scaffolds as well as enhanced growth factor release [17].

For several decades, barrier membranes have been used in oral and maxillofacial surgery. The most popular materials for the manufacture of these membranes are collagen, fresh PCs, and chitosan compounds. Recently, Ansarizadeh [29] reported the integration of chitosan and LPCs into a composite collagen membrane, and the results revealed that both the chitosan/collagen weight percentage and the concentration of LPCs enhanced mechanical strength and membrane biodegradability. Furthermore, increased cell proliferation, higher cell viability, and osteogenic differentiation were enabled by the incorporation of LPCs.

LPCs have also been assessed clinically to determine their applicability as a scaffolding material for other craniofacial tissue regeneration applications [27]. The data demonstrated that constructs exhibited a consistent fibrin structure with a significant leukocyte infiltrate. Transmission electron microscopy (TEM) studies verified the preservation of their potency in promoting tissue healing, even at 1-week following lyophilization at −196 °C. For the assessment of general healing at the surgical site, color, swelling, bleeding, and post-operative pain were assessed to determine the effect of the LPCs. No apparent differences were identified compared with the control group, which was composed of freshly isolated PCs [27]. Consequently, the authors concluded that the LPC constructs promoted bone regeneration and that they also promoted chemotaxis and proliferation of neighboring osteoblast progenitors similarly to the fresh PC isolate control.

Thus far, there have been only a relatively small number of studies analyzing the effects of PCs for pulp regenerative therapies. Two recent reports have, however, aimed at determining the potential application of LPCs in regenerative endodontic procedures [33,34]. The authors reported that the lyophilization approach still enabled comprehensive local release of cytokines. Xu [34], conducting a study on beagle dogs, showed that LPC application was capable of inhibiting the release of pro-inflammatory cytokines while promoting proliferation, migration, and odonto-/osteo-genic differentiation of human dental stem pulp cells (hDPSCs). Furthermore, LPCs also induced dentine–pulp complex regeneration as well as enabling continued root growth in the immature teeth treated [34].

In contrast, a recent study [33] reported that both LPCs and fresh PCs inhibited the mineralization potential during the first week of application. The authors speculated that the stem cells from the apical papilla (SCAP) used in the study were unique compared to other types of mesenchymal stem cells. This is because SCAPs have been certified to regenerate into vascularized dentine–pulp complex in vivo, and it is a stem cell niche that is known to be a source of odontoblasts during dental development. Notably, however, this study still highlighted the potential for application of LPCs in dental tissue engineering, particularly for the treatment of pulp necrosis and periapical periodontitis in immature teeth.

#### 2.2.1. Craniofacial Wound Healing

The interest in the application of tissue engineering and PC strategies for craniofacial wound repair has increased in interest in recent years. Nakajima [35] published a report on a LPC hybrid scaffold preparation using a polyglactin mesh for use as a wound dressing. One of its drawbacks, however, was the increased host inflammatory response that occurred due to the polyglactin degradation. Consequently, the use of a collagen sponge as a carrier for the LPCs has been explored [14]. It was found that the LPC coating improved the collagen sponge’s ability to attract and become infiltrated by fibroblasts, as well as promoting neo-angiogenesis in the surrounding tissue. The data indicated that the LPC-coated collagen sponge exhibited enhanced wound healing and regenerative capacity by stimulation of angiogenesis and infiltration of cells from surrounding tissue without causing a substantial inflammatory response. Furthermore, in the LPC-coated collagen sponge group, they identified considerably thicker capillary blood vessels compared to the noncoated sponge group at 4 weeks and 12 weeks post-implantation. This result is in agreement with Pietramaggiori [36], who noted that LPCs facilitated wound healing in a chronic wound model developed in diabetic mice. Interestingly, in contrast to the nontreated group, this study revealed a significant (*p* < 0.01) fivefold increase in blood vessel density in the LPC group. Recently, Xu et al. [15] suggested that the vascularization process was accelerated in their skin wound study in mice after using a scaffold composed of polyvinyl alcohol (PVA) hydrogel and LPCs as a dressing within 9 days post-surgery. Hence, they believe the neo-angiogenesis could provide oxygen and nutrient to the surgical site, thus promoting protein synthesis.

#### 2.2.2. Lyophilized Platelet Concentrates as a Craniofacial Bioactive Scaffold

Guided tissue repair approaches are dependent upon growth factor activity. Incorporating PCs into a carrier system, such as a 3D scaffold, can enable controlled release of these molecules as well as enhancing their bioavailability. Kutlu [31] demonstrated that a chitosan scaffold loaded with PCs provided an excellent tool for multiple concurrent releases of PC-derived growth factors, and hence recommended its use in tissue repair purposes. Further research has assessed the applicability of LPCs as a scaffold for regeneration of craniofacial tissue and compared their biological effects with fresh PCs [17]. Dental follicle cells, periodontal progenitor cells, and alveolar bone cells were used in comparisons of their cellular activity in response to fresh and lyophilized PCs. The data indicated that LPCs exhibited superior effects compared with fresh PCs as a scaffolding material in terms of induction of proliferation, osteogenic differentiation, and tissue integration [17]. The authors concluded that the LPC preparations not only increased the capacity of cells to migrate and proliferate within the scaffold due to an increased pore size, but the construct also enabled a gradual release of growth factors, such as TGF-β1, PDGF, and VEGF from the biomaterial surface. Similarly, Liu [37] more recently reported that the lyophilization process enhanced the fibrin and platelet structures, enabling improved bioactivity.

As LPCs reportedly continuously release bone regenerating growth factors [13], Li and colleagues [30] compared the utility of 3D-printed polycaprolactone (PCL) scaffolds containing fresh PCs and LPCs for bone repair. The LPC–PCL scaffold exhibited superior stimulation of bone growth as compared with the conventional PC–PCL scaffold. Furthermore, their results indicated that in vivo, mineralization and osteogenesis could be promoted by coating 3D-printed PCL scaffolds with LPCs. The authors postulated that this outcome could be directly linked to the sustained release of PC-derived growth factors, including VEGF, PDGF, basic fibroblast growth factor (bFGF), TGF-β1, EGF, and IGF-1 [30]. Table 2 summarizes the in vitro, in vivo and one randomized clinical trial application of LPCs to enhance tissue regeneration. Table 3 summarises the strengths and limitations of LPC.

#### 2.2.3. Limitations of the Study

The dynamic biology of PCs and their role in tissue regeneration and inflammation have provided the basis for PC and LPC therapies for a wide variety of medical and dental treatments. However, scientific advancements remain hindered by the lack of standardization of PC and LPC products, doses, and fabrication protocols [38]. The situation continues to be ambiguous as the different approaches and materials used do not seem to produce the same material as original PCs. Moreover, heterogeneous processing methods, unstandardized nomenclature, and vague classifications complicate comparisons among studies; thus, we recommend a comprehensive, detailed and step-by-step explanation of the preparation procedure for the LPC to allow for comparison between studies, ensuring reproducibility to prevent misunderstanding and misleading assumptions in the literature (Table 3) [38,39,40,41].

## 3. Materials and Methods

**Figure 3:**
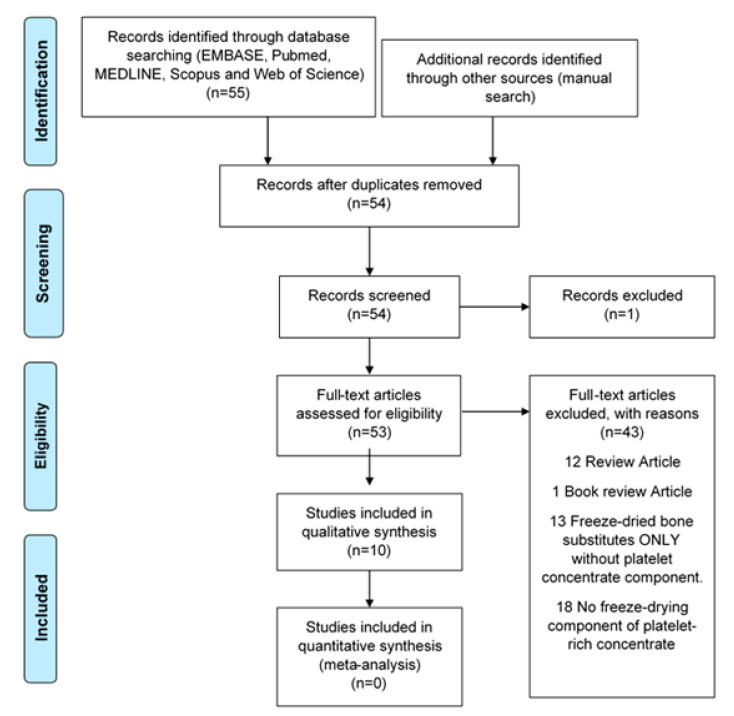
Flowchart of manuscript screening process and inclusion in the review.

The relevant literature was searched for electronically up to 30 September 2019 to identify reports evaluating LPCs in EMBASE, PubMed, MEDLINE, Scopus, and the Web of Science. The following keywords were used for MEDLINE and EMBASE as MESH terms or free words: (Platelet-rich plasma OR platelet-rich fibrin OR platelet concentrate* OR concentrated growth factor*) AND (Freeze-drying OR lyophilisation OR lyophilization) AND (Craniofacial OR facial bone* OR face* OR oral OR dentist*) AND (Bone regeneration OR tissue regeneration OR tissue engineering). Publications were identified from citations of selected papers to improve search sensitivity. Only studies published using the English language were included, and duplicates were eliminated (Figure 3).

Selected research papers describing (1) the outcomes of lyophilization of PCs; (2) the preservation status of growth factors after the lyophilization process; (3) the biological properties of LPCs in in vitro and in vivo models; and (4) novel directions for LPC research in craniofacial regeneration were included.

The information provided in this review indicates that lyophilization provides a potential benefit for PCs for use in craniofacial tissue regeneration approaches. This is due to the increased preservation of growth factor bioactivity following freeze-drying due to its transformation of the fibrin and PC structure. Combined, these characteristics of LPCs for use in clinical practice extend shelf-life, enable emergency medicine usage, improve sample storage, and facilitate transportation management. However, despite these improvements, research is still required to improve the mechanical properties of LPCs as well as providing efficient delivery and release systems.

Nevertheless, the drawbacks of lyophilization include fabrication cost and the possibility of LPCs contamination. Additionally, a standardization protocol of all PC products has to be addressed, including standardizing the temperature, duration of lyophilization, and storage of the material [37]. In light of this, further preclinical studies and clinical trials are still required to enable LPCs to be routinely applied in craniofacial tissue regenerative therapies.

## Figures and Tables

**Table 1:** Different preparation methods of lyophilized platelet concentrates as craniofacial bioactive scaffolds.

No.	Type	Platelet Concentrate Preparation Protocol	Fabrication of Composite Scaffold with Lyophilized Platelet Concentrates	Scaffold	Cell Type	Animal Model	In Vitro/In Vivo Analysis Method	Main Findings	Author, Year, References
1.	PC	10 mL of plasma centrifuged at 4450 rpm for 10 min.	The PC solutions were frozen for one hour in a −20 °C freezer and then for 2 h in a −80 °C until 24 h freezing. Then, the fabricated FDPC powder was combined with the chitosan mixture and β-GP.	Thermo-sensitive chitosan/β-glycerol phosphate (β-GP) hydrogel.	PDLSCs	NA	In vitro	FDPC-loaded hydrogel groups show two weeks of continuous release of TGF-β1 and PDGF-BB. The growth factor release profiles exhibited a similar pattern.	Ammar 2018 [28]
2.	A-PRF	10 mL of blood centrifuged at 1500 rpm for 14 min.	The solutions for collagen and chitosan were blended and cross-linking before being agitated for 24 h. At −80 °C, the PC was frozen and dried at −40 °C for 24 h. The lyophilized PC was supplemented to the solution, immediately cast, frozen, and freeze-dried.	Collagen–chitosan membrane with Lyophilized A-PRF.	MSCs	NA	In vitro	A-PRF lowered the rate of degradation and Young’s modulus of the scaffold. A-PRF induced better cell viability and osteogenic differentiation compared to the control group.	Ansarizadeh 2019 [29]
3.	PRP	Blood centrifuged at 2400 and 3600 rpm for 10 and 15 min.	For 5 min, PCL scaffolds were submerged in PRP at RT and then stored at −80 °C for 30 min. The frozen samples were immediately freeze-dried. Pending use, the FD-PRP-PCL scaffold was stored at 4 °C.	Traditional PRP–PCL scaffolds, bare PCL scaffolds and, the freeze-dried PRP–PCL scaffolds.	DPSCs	Rats	In vitro In vivo	FD-PRP stimulated ALP, RUNX2, OCN and OPN mRNA expression. Scaffolds of the FD-PRP-PCL caused more significant bone formation.	Li 2017 [30]
4.	PRP	NA	A collagen sponge was dipped in PRP. The PRP-absorbed collagen sponge was frozen for 60 min at −75 °C and freeze-dried later. It was then kept at 4 °C until used.	FD-PRP-coated collagen sponge with a non-FD-PRP coated collagen sponge.	hAPCs	Mice	In vitro In vivo	PRP-coated sponge failed to induce hAPC proliferation. PRP-coated sponge rapidly caused angiogenesis and the invasion of the connective tissue around it.	Horimizu 2013 [14]
5.	PRP	8.5 mL of blood centrifuged at 2400 rpm (103 g) and 3600 rpm (230 g) for 10 and 15 min.	GEL scaffold: PRP was added to chitosan gel and then freeze-dried. SPONGE scaffold: PRP was implanted to freeze-dried chitosan scaffolds using a micropipette.	GEL and SPONGE chitosan scaffold.	NA	NA	In vitro	In the GEL group, a continuous release of GFs was achieved, while a rapid burst release was detected in the SPONGE groups. GEL scaffolds had their porous structure preserved. The GEL scaffold is superior to the SPONGE scaffold because of the morphological architecture of the scaffold.	Kutlu 2013 [31]

PC: platelet concentrates; FDPC: freeze-dried platelet concentrate; β-GP: β-glycerol phosphate; PDLSCs: periodontal ligament stem cells; TGF-β1: transforming growth factor β-1; PDGF-BB: platelet-derived growth factor-BB; A-PRF: advanced platelet-rich fibrin; MSCs: mesenchymal stem cells; PRP: platelet-rich plasma; PCL: polycaprolactone; FD-PRP: freeze-dried platelet-rich plasma; FD-PRP-PCL: freeze-dried platelet-rich plasma and polycaprolactone; DPSCs: dental pulp stem cells; ALP: alkaline phosphatase; RUNX2: runt-related gene-2; OCN: osteocalcin; OPN: osteopontin; PDLSCs: periodontal ligament stem cells; hAPCs: human alveolar periosteal cells; GFs: growth factors; NA: not available.

**Table 2:** Summary findings of in-vitro, in-vivo and clinical trial studies using lyophilized platelet concentrates (LPCs).

No.	Type	Platelet Concentrate Preparation Protocol	Lyophilization Method	Comparison Group	Cell Type	Animal Model	Type of Study	Main Finding	Author, Year, References
1.	PRF	8 mL blood centrifuged at 1700 rcf for 5 min.	The PRF membrane was frozen for 30 min at −80 °C and freeze-dried overnight (−54 °C, 12 Pa).	Fresh PRF and frozen PRF	MSCs, HGFs	NA	In vitro	In FD-PRF, the proliferation of MSCs was greater. Frozen PRF and FD-PRF were more compact and had a rough texture. Frozen PRF had lower activity in plasmin.	Kardos 2018 [32]
2.	PRF	10mL blood centrifuged at 2100 rpm (400 g) for 12 min.	The frozen PRF membranes were kept at −80 °C. The frozen PRF was then freeze-dried at −51 °C overnight.	Fresh PRF	DFs, Abs, PDLs	Rats	In vitro In vivo	L-PRF caused the proliferation and migration of the PDL cells. In AB cells, L-PRF stimulated RUNX2. L-PRF protected 97% of bone defects compared to 84% in the case of fresh PRF.	Li 2014 [17]
3.	CGF and PRF	10 mL of blood.	In a vacuum freeze dryer, the CGF and PRF membranes were frozen overnight.	FD-PRF and FD-CGF	SCAPs	NA	In vitro	Major growth rate and migratory cells in FD-CGF and FD-PRF groups. After 7 days and 14 days, substantial mineralized areas in FD-CGF and FD-PRF.	Hong 2018 [33]
4.	CGF	10 mL blood centrifuged for 2 min at 2700 rpm (600 g), 4 min at 2400 rpm (400 g), 4 min at 2700 rpm (600 g), and 3 min at 3000 rpm.	The isolated CGF membranes were frozen in a vacuum freeze dryer overnight.	NA	hDPSCs	Beagle dogs	In vitro In vivo	CGF had a protective effect on the inflamed hDPSCs. CGF had a strong impact on hDPSC proliferation, migration. and differentiation. CGF facilitated complex regeneration of the dentine pulp in immature teeth.	Xu 2019 [34]
5.	L-PRF	10 mL of blood centrifuged at 3000 rpm (400 g) for 10 min.	5% DMSO and PRF were freeze-dried for 24 h at −80 °C and cryopreserved for a week in the −196 °C. L-PRF was thawed for 3 min, then rinsed with PBS two to three times. Later, L-PRF was fixed in 4% paraformaldehyde.	Fresh PRF and L-PRF were implanted into the patient’s edentulous anterior maxillary region for GBR.	NA	NA	Clinical trial	Fresh PRF and L-PRF illustrated clinical and immunohistochemical similarities. L-PRF growth factors and fibrin networks were able to facilitate chemotaxis and the proliferation of adjacent osteoblasts.	Zhang 2017 [27]

PRF: platelet-rich fibrin; FD-PRF: freeze-dried PRF; L-PRF: lyophilized-platelet-rich fibrin, FD-CGF: freeze-dried concentrated growth factors, CGF: concentrated growth factor; MSCs: mesenchymal stem cells; HGFs: human gingival fibroblasts; DF; dental follicle; AB: alveolar bone; PDL: periodontal ligament; SCAPs: stem cells from apical papilla; hDPSCs: human dental stem pulp cells; RUNX2: runt-related transcription factor2; DMSO: dimethyl sulfoxide, PBS: phosphate buffer solution; GBR: guided bone regeneration, NA: not applicable.

**Table 3:** Strengths and limitations of the lyophilized platelet concentrate (LPC) [38].

**Strength**	1. Preservation of biological properties;
	2. Preservation of morphological architecture;
	3. Sustained release of growth factors;
	4. 100% natural and autologous;
	5. Biocompatible with other biomaterials;
	6. Multiple usages with single venipuncture;
	7. Easy Transportation;
	8. Better storage capabilities;
	9. Enables use in emergency surgery;
	10. Longer clinical shelf-life.
**Limitation**	1. Fabrication cost;
	2. Possible risk of contamination;
	3. Demands standardization protocol for lyophilization technique.

## Data Availability

The data presented in this study are available on request from the corresponding author.
